# Predominant SARS-CoV-2 variant impacts accuracy when screening for infection using exhaled breath vapor

**DOI:** 10.1038/s43856-022-00221-5

**Published:** 2022-12-08

**Authors:** Mitchell M. McCartney, Eva Borras, Dante E. Rojas, Tristan L. Hicks, Katherine L. Hamera, Nam K. Tran, Tina Tham, Maya M. Juarez, Enrique Lopez, Nicholas J. Kenyon, Cristina E. Davis

**Affiliations:** 1 Mechanical and Aerospace Engineering, UC Davis, Davis, CA USA; 2 UC Davis Lung Center, Davis, CA USA; 3 VA Northern California Health Care System, Mather, CA USA; 4 Department of Pathology and Laboratory Medicine, UC Davis, Sacramento, CA USA; 5 Department of Internal Medicine, UC Davis, Sacramento, CA USA; 6 UC Davis School of Medicine, Sacramento, CA USA

**Keywords:** Infectious diseases, Diagnostic markers, Diagnosis, Metabolomics

## Abstract

**Background:**

New technologies with novel and ambitious approaches are being developed to diagnose or screen for SARS-CoV-2, including breath tests. The US FDA approved the first breath test for COVID-19 under emergency use authorization in April 2022. Most breath-based assays measure volatile metabolites exhaled by persons to identify a host response to infection. We hypothesized that the breathprint of COVID-19 fluctuated after Omicron became the primary variant of transmission over the Delta variant.

**Methods:**

We collected breath samples from 142 persons with and without a confirmed COVID-19 infection during the Delta and Omicron waves. Breath samples were analyzed by gas chromatography-mass spectrometry.

**Results:**

Here we show that based on 63 exhaled compounds, a general COVID-19 model had an accuracy of 0.73 ± 0.06, which improved to 0.82 ± 0.12 when modeling only the Delta wave, and 0.84 ± 0.06 for the Omicron wave. The specificity improved for the Delta and Omicron models (0.79 ± 0.21 and 0.74 ± 0.12, respectively) relative to the general model (0.61 ± 0.13).

**Conclusions:**

We report that the volatile signature of COVID-19 in breath differs between the Delta-predominant and Omicron-predominant variant waves, and accuracies improve when samples from these waves are modeled separately rather than as one universal approach. Our findings have important implications for groups developing breath-based assays for COVID-19 and other respiratory pathogens, as the host response to infection may significantly differ depending on variants or subtypes.

## Plain language summary

In recent decades, scientists have found we exhale thousands of compounds that reveal much about our health, including whether we are sick with COVID-19. Our team asked whether the breath profile of someone infected with the Delta variant of COVID-19 would match the breath profile caused by the Omicron variant—a version of the virus that is more transmissible. We analyzed breath samples from 142 people, some sick with either the Delta or Omicron variant of COVID-19, and others who were negative for COVID-19. Our results indicate that the Delta variant altered the contents of our breath in a different way than the Omicron variant, and breath-based tests improved when optimized to detect only one of the variants. These findings might impact the design of future breath-based tests for COVID-19.

## Abstract (web summary)

McCartney, Borras et al. analyse the metabolites present in breath samples collected from people with or without COVID-19. The authors find that the breath metabolite signature differs based on the predominant SARS-CoV-2 variant circulating at the time, and that breath test accuracy improves when modelling samples from each variant wave separately.

## Introduction

The coronavirus disease 2019 (COVID-19), caused by severe acute respiratory syndrome coronavirus 2 (SARS-CoV-2), has caused an ongoing pandemic. Subsequently, new diagnostic tests and screening tools have been established for both clinical and at-home use to augment the nasal and oropharyngeal swab procedures coupled with a reverse transcription polymerase chain reaction (RT-PCR). Often, alternatives seek to increase the speed that results are obtained and diversify reagents and sampling materials to alleviate stress on global supply chains.

Institutions worldwide have heavily invested in the development of novel—sometimes ambitious—assays and technologies that diagnose or screen for SARS-CoV-2. In 2020, the National Institutes of Health in the United States launched the RADx℠ Radical program to “support innovative, non-traditional diagnostic approaches to address gaps in COVID-19 testing and surveillance” (www.radxrad.org). The 8 focus areas of RADx-rad include the SCENT program to develop and commercialize new technology platforms to screen for COVID-19 from metabolites found in exhaled breath.

Breath is an opportunistic biospecimen to diagnose or screen for SARS-CoV-2 infections as it can be collected non-invasively and is readily available^1^. While some portions of breath research can directly identify SARS-CoV-2 virus or proteins exhaled by infected persons, most measure the hundreds to thousands of endogenous human metabolites found in breath and train statistical models to identify the host response to infection.

Few breath tests have received regulatory approval, such as the ethanol breathalyzer or urea breath test for *Helicobacter pylori*. However, the US Food and Drug Administration approved the first COVID-19 diagnostic test based on exhaled breath metabolites on April 14, 2022 under emergency use authorization (EUA). After validation in a large study of 2409 individuals, the test has a sensitivity of 91.2% and 99.3% specificity^2^. It is anticipated that the FDA and other international regulatory agencies will consider other breath tests to screen for SARS-CoV-2 infections as new technologies emerge.

Two major breath fractions can be collected. Exhaled breath condensate is the condensation of exhaled aerosols and contains larger, non-volatile metabolites^3,4^ which can be used to assess pulmonary health and disease state^5,6^. Because it is comprised of aerosol droplets, exhaled breath condensate is a known vector for SARS-CoV-2 transmission^7^, though deactivation procedures have been reported for safe clinical handling^8^.

Exhaled breath vapor is the collection of gas-phase compounds, including respiratory gases like O_2_, CO_2_, and NO, but also hundreds of volatile organic compounds (VOCs) known as the breath volatilome^9^. While exogenous compounds are the result of inspiring VOCs from the room or environment, endogenous compounds derive from metabolic processes and can reflect imbalances due to injury or disease^10^.

Clinical practices have been developed to safely collect, handle, and deactivate potentially transmissible viral material in breath vapor samples for COVID-19 screening^11–13^. Worldwide, breath research groups have shown that exhaled vapor reflects SARS-CoV-2 infection status^14–16^. Breath biomarkers have even been established to distinguish SARS-CoV-2 infection in children^17^. As with other breath researchers, we hypothesized that a profile of metabolites in exhaled breath vapor could distinguish patients with SARS-CoV-2 infection from uninfected patients. However, we observed a shift in the breathprint of infection as the dominant variant of transmission shifted in our community.

As far as we know, we are the first to detail how the exhaled breath vapor profile differs in those infected during the Delta (B.1.617.2) versus Omicron (B.1.1.259) waves as measured by thermal desorption-gas chromatography-mass spectrometry (TD-GC-MS). Validated statistical models have increased accuracies when modeling the variant waves independently, rather than creating a universal COVID-19 model that does not consider the dominant variant. These findings have significant implications for teams developing breath-based diagnostics or screening tools for SARS-CoV-2.

## Methods

### Participant recruitment

Recruitment of 142 volunteers and sample collection was conducted under a previously approved protocol for human subjects research (UC Davis IRB #1636182). We recruited persons aged 18 and older from the UC Davis campus, its medical center and surrounding clinics, and from the greater Sacramento community. All participants signed informed consent and then completed a questionnaire to obtain demographic data, such as age, race, ethnicity, COVID-19 vaccination status, medical history, symptoms, and more. Volunteers then provided a breath sample (procedure below) and were compensated with a $25 gift card for their time.

### Confirmatory SARS-CoV-2 testing

Not all participants were tested for SARS-CoV-2 at the time of breath collection. Those recruited into the COVID(+) cohort were either hospitalized for ongoing COVID-19 treatments and had recently tested positive for SARS-CoV-2, or were pending a COVID-19 test result at the time of collection that subsequently returned as positive. SARS-CoV-2 tests were all PCR-based. Subjects were either tested as part of their clinical treatment for COVID-19 at the UC Davis Medical Center or were voluntarily testing due to respiratory symptoms at the Healthy Davis Together community site, a COVID-19 testing operation open to the community and funded by both UC Davis and the City of Davis, California. Inclusion criteria for participants recruited for the COVID(−) cohort were that they were either: (a) asymptomatic with no known exposure to the virus and had not received a positive test result within 2 weeks prior, or (b) were symptomatic at time of collection and pending a COVID-19 test result subsequently returned as negative.

COVID-19 status was determined by SARS-CoV-2 RNA detection by FDA emergency use authorized RT-PCR tests. At our institution, RT-PCR testing was performed via the high-throughput cobas 6800/8800 SARS-CoV-2 assay (Roche Molecular Systems). Patients admitted with COVID-19 from the Emergency Department are confirmed by the cobas Liat SARS-CoV-2 assay (Roche Molecular Systems). One Delta-wave subject had their COVID positive status confirmed by a loop-mediated isothermal amplification test rather than PCR.

### Paired breath and environmental VOC collection

Briefly, participants exhaled into Tedlar bags. The volatile contents of the breath samples were loaded from the bags onto sorbent-packed tubes and then chemically analyzed using thermal desorption-gas chromatography-mass spectrometry. A corresponding background environmental VOC sample was also collected to remove exogenous artefacts. A full description follows.

For VOC analysis, Tenax TA packed thermal desorption tubes were used (Gerstel Inc, #020810-005-00). Before each use, tubes were conditioned at 280 °C for 15 min under a flow of helium and analyzed using the same method described below to ensure tubes were void of contaminant volatiles.

Volunteers were asked to not eat or drink 1 h prior to breath collection. No nose clip or mouth rinse was used. Volunteers were instructed to fill a 5 L Tedlar bag (Millipore Sigma SKU 24655) using normal, tidal breathing. After collection, the sample was connected to a VOC extraction platform previously described^18^ that was designed to load gas-phase samples onto sorbent-packed tubes for subsequent thermal desorption analysis. Briefly, the VOC extractor was calibrated daily to a flow rate of 250 mL/min. Exactly 2 L of exhaled breath vapor was extracted onto a sorbent-packed tube.

Paired background VOC samples were collected by inserting a second thermal desorption tube into the sampler, which extracted 2 L of the room or environment air. At least one background sample was collected to represent no more than 3 participants collected within 1 h and within the same room or location.

After extraction, an aliquot of a first internal standard, chlorobenzene-d5, was added to each sorbent tube (1 µL of 23.11 ppm) and refrigerated until analysis. This first internal standard was intended to monitor any error resulting from storage and subsequent instrument analysis of each sample. Samples were typically analyzed within 2–3 days of collection and never stored for more than 5 days. Just before analysis, samples were spiked with a second internal standard, naphthalene-d8 (1 µL of 500 ppb). This second internal standard was intended to monitor only any error resulting from the instrumental analysis of each sample. The internal standard response for every sample was inspected prior to any statistical analysis to screen for potential outliers due to sample handling or instrumental analysis; no such issues were found and no samples were removed from the dataset.

### Thermal desorption-gas chromatography-mass spectrometry (TD-GC-MS) analysis

Samples underwent TD-GC-MS analysis with a Gerstel TDU2 thermal desorption unit, CIS4 cryofocusing unit and MPS autosampler system. The thermal desorption temperature started at 30 °C and held for 3 min, then ramped 300 °C/min to a final temperature of 280 °C, holding for 5 min. Desorbed volatiles were led by carrier gas via a transfer line set to 280 °C to the cryofocusing CIS, which held at −100 °C during desorption. After desorption was complete, the CIS4 splitlessly injected volatiles onto the column by ramping at 12 °C/s to 280 °C, and held for 3 min. For chromatography, an Agilent 7890 A GC was used, equipped with a DB-5ms column (30 m × 250 µm × 0.25 µm, Agilent Technologies Inc.). The column oven was initially set to 38 °C for 3 min, then ramped at 3 °C/min to 110 °C, then 5 °C/min to 170 °C for 1 min, then 20 °C/min to 280 °C for 4.5 min, for a total run time of 50 min. Constant flow mode was used with 1.8 mL/min of ultra-high purity helium. VOCs were detected with an Agilent 5975 C single quadrupole mass spectrometer, which scanned an *m/z* range of 30–350, with source temperature 230 °C and quadrupole temperature 150 °C.

Sorbent tube blanks, breath samples and corresponding environmental samples were randomly injected on the instrument to obtain reliable data. Alongside every batch of samples, a Grob mix was injected in triplicate to monitor instrument performance, and a Kovats mixture of C_7_–C_30_ alkanes was injected to monitor retention time drift and to calculate Kovats retention indices of measured VOCs. TD-GC-MS system blanks were collected every 20 sample injections to ensure the system was clear of artefacts.

Putative compound identifications were completed by comparing the obtained mass spectra to the NIST 2020 database and by comparing the obtained Kovats Index for each compound to indices reported in the literature.

### Statistics and reproducibility

Raw mass spectral data were deconvoluted and aligned using Agilent Profinder B.08 and Mass Profiler Professional (MPP, V13.0). Statistical analyses were performed using Mathworks MATLAB R2022a and Eigenvector Research’s PLS_Toolbox version 9.0. Data were normalized by a log_10_ transformation, followed by Pareto scaling and mean-centering.

GC-MS analysis of 408 sorbent tube blank injections and breath/environmental samples resulted in a table of 530 features/variables. Data were cleaned by removing non-informative features and background environmental artefacts. First, system blank (no sample injection) and sorbent tube blank (clean sorbent tubes) features that appeared with a sample:blank ratio less than 3 were removed, as well as features missing in more than 25% of samples. Then, features appearing in both breath samples and their corresponding environmental VOC samples were removed when the breath:background ratio was lower than 2. These filtering resulted in an initial dataset containing 142 breath samples and 108 variables.

Variables were further filtered to those that may discriminate COVID(+) and COVID(−) samples. Each model (Delta + Omicron, Delta-only, Omicron-only) was built using partial least squares-discriminant analysis in 50 iterations. All iterations were calibrated on an equal ratio of COVID and non-COVID samples, using a random 66% of samples from the group with fewer samples. Models were then validated with the remaining samples. From these initial models, a total of 63 VOCs had a variance in projection (VIP) score 1. Using only these 63 compounds, final models (Delta + Omicron, Delta-only, Omicron-only) were rebuilt using the same 50 iteration fashion and random 66% calibration strategy. Values resulting from these final models (VIP scores, accuracies, etc.) are presented in the following Results and discussion section.

### Reporting summary

Further information on research design is available in the Nature Portfolio Reporting Summary linked to this article.

## Results and discussion

Demographic information for participants enrolled in this study is presented in Table 1. Because a portion of enrollment occurred at a community testing site for those experiencing symptoms, 31.3% of non-COVID control participants self-reported respiratory symptoms at the time of breath collection. 90.6% of non-COVID control subject had at least one vaccine dose at the time of collection, whereas only 22.3% of the Delta-wave cohort but 82.2% of the Omicron wave cohort were vaccinated.

**Table 1:** Demographic information.

	Delta	Omicron	Non-COVID controls
Number of subjects	12.7% (18)	19.7% (28)	67.6% (96)
Respiratory symptoms at time of sampling^a^	55.6% (10)	82.1% (23)	31.3% (30)
Age (mean ± standard deviation)	49 ± 19	45 ± 22	40 ± 18
Race			
American Indian or Alaska Native	0.0% (0)	0.0% (0)	1.0% (1)
Asian or Pacific Islander	0.0% (0)	10.7% (3)	13.5% (13)
Black or African American	11.1% (2)	14.3% (4)	6.3% (6)
Caucasian	72.2% (13)	57.1% (16)	55.2% (53)
Other	16.7% (3)	17.8% (5)	22.9% (22)
Multi-racial	0.0% (0)	0.0% (0)	1.0% (1)
Hispanic, Latino, or Spanish origin	11.1% (2)	17.8% (5)	29.2% (28)
Biological sex			
Female	55.6% (10)	64.3% (18)	58.3% (56)
Male	44.4% (8)	34.7% (10)	41.7% (40)
Gender identity			
Female	55.6% (10)	64.3% (18)	56.3% (54)
Male	44.4% (8)	35.7% (10)	43.8% (42)
Vaccination status			
No doses	77.8% (14)	17.9% (5)	9.4% (9)
1 dose	0.0% (0)	3.6% (1)	2.1% (2)
2 doses	16.7% (3)	39.3% (11)	45.8% (44)
3+ doses	5.6% (1)	39.3% (11)	42.7% (41)

Percentages are reported with total numbers in parentheses.

^a^Subjects that self-reported having at least one of the following at time of breath collection: cough, shortness of breath or difficulty breathing, new loss of taste or smell, sore throat.

PCR samples from COVID(+) participants were not sequenced and thus we were unable to confirm the SARS-CoV-2 variant of infection. However, based on sequencing surveillance data of COVID(+) patients from the UC Davis Health Emergency and genotyping surveillance data from Healthy Davis Together (Table 2), our community shifted from the Delta variant to Omicron during the month of December 2021. COVID instances in our region were only 3–20% Omicron the week of 12/06/2021, but Omicron quickly accelerated to 98–100% of infections by 01/03/2022. The date that each breath sample was collected is provided in Supplementary Data 1. Six COVID(+) breath samples were collected in the month of December 2021, all during the week of 12/13/2021. During that time, Omicron was between 17 and 43% of COVID(+) cases in our community. Thus, we could not confidently presume the variant of infection for these six breath samples, and they were removed from the dataset, reducing the total number of samples to 136 (from 142). Samples from COVID(+) subjects collected through November 24, 2021 were presumed to be Delta-infected, and samples collected after January 11, 2022 were presumed to be Omicron-infected.

**Table 2:** Sequencing surveillance data from the UC Davis Health (UCDH) and genotyping surveillance data from Healthy Davis Together (HDT), showing when the Omicron variant overwhelmed Delta among COVID(+) patients in our region.

Week of	12/06/2021		12/13/2021		12/20/2021		12/27/2021		01/03/2022	
	UCDH	HDT	UCDH	HDT	UCDH	HDT	UCDH	HDT	UCDH	HDT
Delta	80% (4)	97% (71)	83% (10)	57% (62)	55% (6)	20% (57)	20% (3)	5% (41)	0% (0)	2% (8)
Omicron	20% (1)	3% (2)	17% (2)	43% (46)	45% (5)	80% (222)	80% (12)	95% (835)	100% (5)	98% (433)

Percentages are reported with total numbers of analyzed samples in parentheses.

**Fig. 1:**
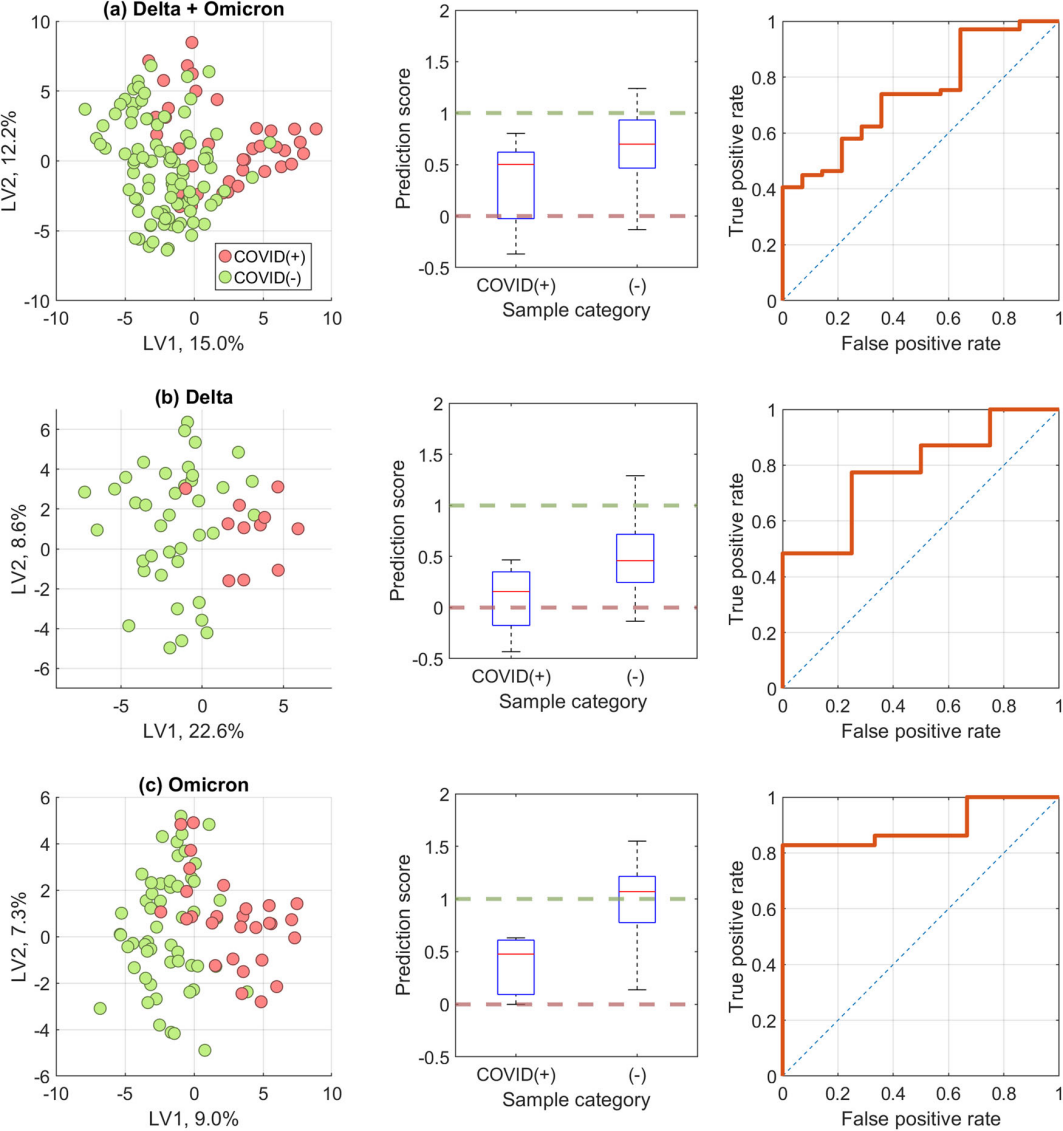
Comparisons of breath-based models calibrated with and without regard to COVID variant. Partial least squares-discriminant analysis (PLS-DA) model comparisons when models were developed using **a** breath samples collected during the wave of both variants, **b** just the Delta wave, and **c** just the Omicron wave. Data show scatter plots of latent variable (LV) scores; boxplots of PLS-DA prediction scores from breath samples with 1 modeled as COVID(−) and 0 as COVID(+); and receiver operator characteristics (ROC) curves showing model accuracies to predict COVID infection from breath volatile organic compounds (VOCs). Analyses were conducted from breath samples of *n* = 96 COVID(−), *n* = 12 Delta COVID(+), *n* = 28 Omicron COVID(+) persons.

Figure 1 shows the results of partial least squares-discriminant analysis (PLS-DA) models calibrated and validated from breath samples. Three separate models were built using samples from (A) both waves, (B) samples from the Delta wave, and (C) samples from the Omicron wave. Each model underwent 50 iterations with a randomized calibration and validation dataset. The accuracies, sensitivities, and specificities are provided in Table 3.

**Table 3:** PLS-DA results showing mean ± standard deviation values for validation sets after 50 iterations of each model, built on the 63 VOCs presented in Supplementary Data 2.

	Delta + Omicron waves	Delta wave	Omicron wave
Accuracy	0.73 ± 0.06	0.82 ± 0.12	0.84 ± 0.06
Sensitivity	0.70 ± 0.08	0.71 ± 0.12	0.78 ± 0.08
Specificity	0.61 ± 0.13	0.79 ± 0.21	0.74 ± 0.12

The overall accuracies were higher when the waves of variants were modeled independently, rather than when combined into one universal model. An Omicron wave model had the highest overall accuracy, 0.84 ± 0.06, followed by Delta (0.82 ± 0.12) and the universal model (0.73 ± 0.06). Per a one-way analysis of variance or ANOVA (*p* = 1.73 × 10^−8^), the universal model accuracy was significantly different than the Delta- and Omicron-only models.

The Delta wave and Omicron wave models had sensitivities, or true positive proportions, of 0.71 ± 0.12 and 0.78 ± 0.08, respectively, whereas universal model sensitivity was 0.70 ± 0.08. Per ANOVA (*p* = 3.67 × 10^−5^), the universal and Delta model sensitivities did not significantly differ between each other but were both significantly lower than the Omicron-only model. The specificity, or true negative proportions were not significantly different between the Delta (0.79 ± 0.21) or the Omicron (0.74 ± 0.12) waves but were significantly different from universal model (0.61 ± 0.13) per ANOVA (*p* = 4.61 × 10^−7^).

Of the symptomatic non-COVID subjects, 90% were correctly identified as COVID(−) in the Delta + Omicron model, which decreased to 33% in the Delta-wave model. However, 64% of symptomatic COVID(−)s were correctly identified in the Omicron model. These findings suggest that Omicron was more accurately identified from other respiratory infections or symptoms, relative to the Delta-only models. Additional work is needed to both train models to identify breathprints of COVID infection apart from other common respiratory pathogens, such as influenzae or rhinoviruses, to determine the selectivity of breath-based test for SARS-CoV-2. More on this is discussed below.

In December 2021, the Omicron variant (B.1.1.529) quickly became the dominant variant circulating worldwide, replacing the Delta variant (B.1.617.2). Numerous Omicron subvariants have now arisen, which have enabled researchers to follow the genetic mutations in this complex coronavirus, as well as the varying clinical manifestations. All Omicron variants are significantly more transmissible and evade primary-series vaccination^19^. There is less infection of airway epithelial cells of the lower respiratory tract, shorter illness periods and generally causes less severe disease than previous variants^20^. Because exhaled breath metabolites can reflect the cascade of thousands of biochemical metabolic processes related to infection, particularly in the lower airway compartment, it is likely the breathprint of COVID infection differ measurably between variants. There is limited understanding of this drift in exhaled metabolites, to date.

**Fig. 2:**
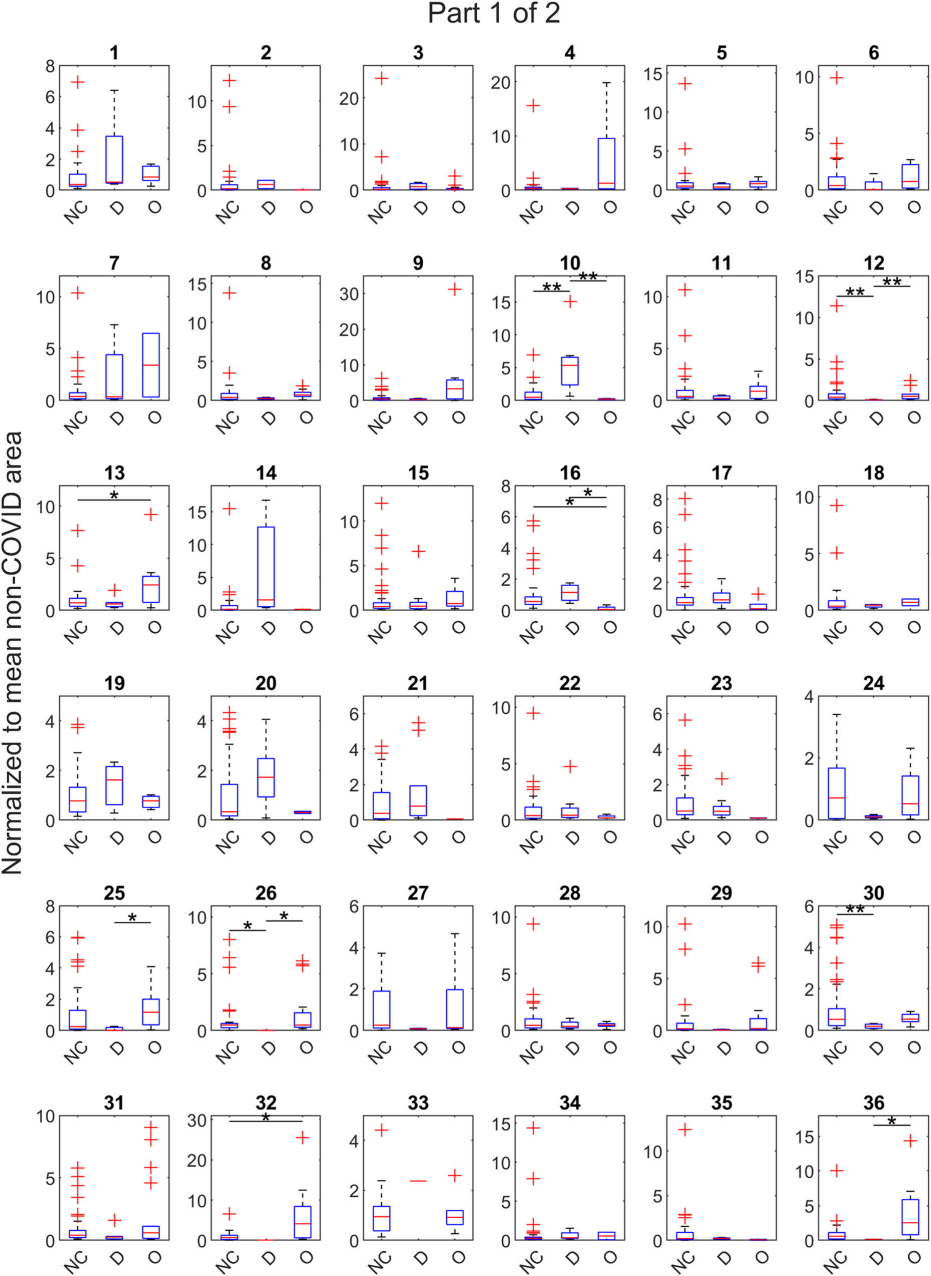
Abundances of exhaled volatile compounds considered by breath-based models to predict COVID-19 infection (see also Fig. 3). Boxplots representing the abundances of the 63 volatile organic compounds (VOCs) (continued in Fig. 3) used by partial least squares-discriminant analysis (PLS-DA) models to differentiate breath samples of non-COVID (NC) controls, *n* = 96, from COVID(+) samples collected during the Delta (D), *n* = 12, and Omicron (O), *n* = 28, waves. For each compound, the data were normalized to the mean intensity from non-COVID controls, so *y*-axis values represent the number of times greater relative to the mean non-COVID abundance. Asterisks indicate significant differences per a Kruskal–Wallis test between two groups (*p < 0.05, **p < 0.01). See Supplementary Data 2 to map compound number to putative identification. The central red line indicates median, bottom and top edges indicate 25th and 75th percentiles respectively, whiskers extend to the most extreme non-outlier data points, and outliers are plotted with the red ‘+’ symbol.

**Fig. 3:**
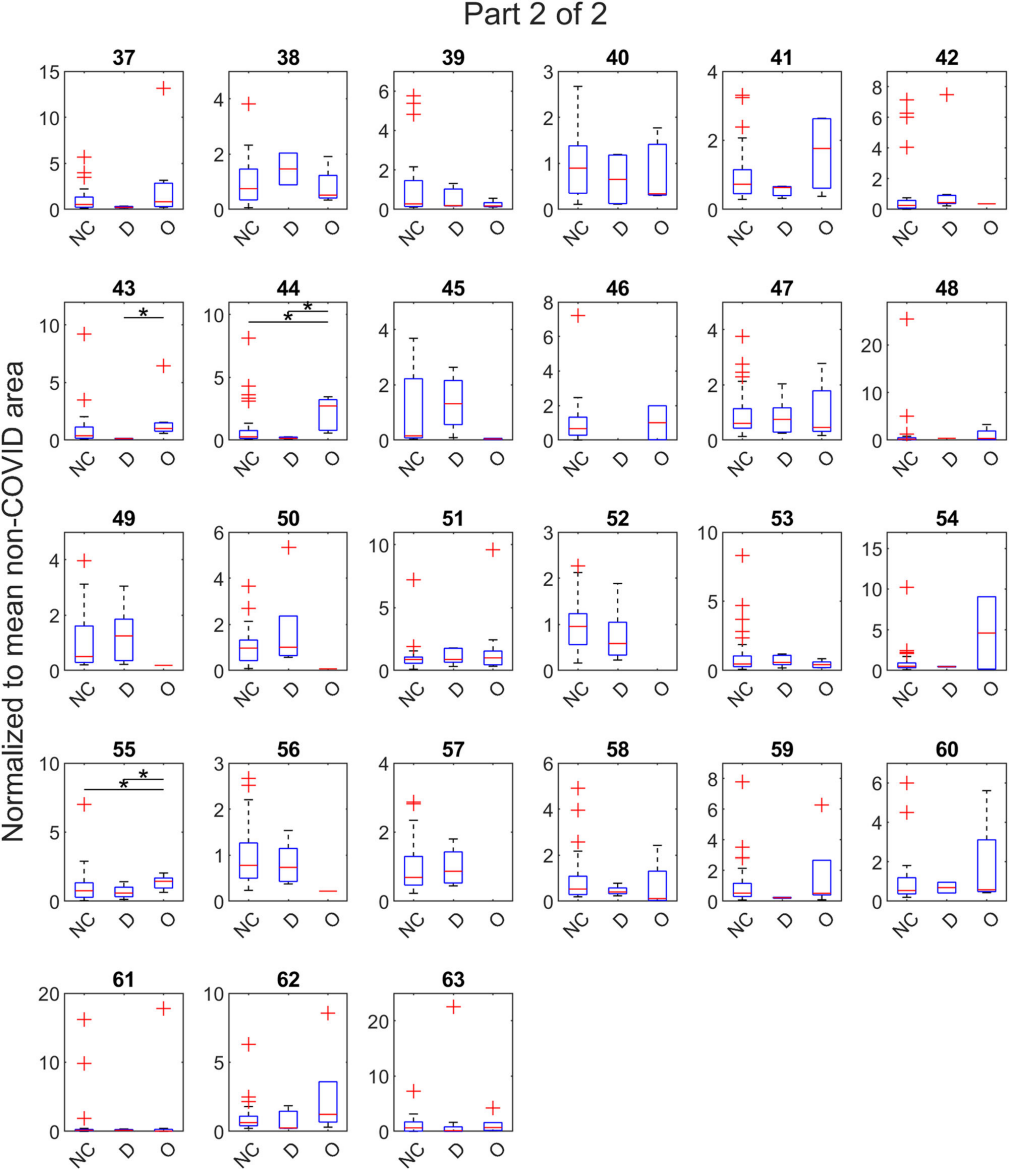
Abundances of exhaled volatile compounds considered by breath-based models to predict COVID-19 infection (see also Fig. 2). Boxplots representing the abundances of the 63 volatile organic compounds (VOCs) (continued in Fig. 2) used by partial least squares-discriminant analysis (PLS-DA) models to differentiate breath samples of non-COVID (NC) controls, *n* = 96, from COVID(+) samples collected during the Delta (D), *n* = 12, and Omicron (O), *n* = 28, waves. For each compound, the data were normalized to the mean intensity from non-COVID controls, so *y*-axis values represent the number of times greater relative to the mean non-COVID abundance. Asterisks indicate significant differences per a Kruskal–Wallis test between two groups (*p < 0.05, **p < 0.01). See Supplementary Data 2 to map compound number to putative identification. The central red line indicates median, bottom and top edges indicate 25th and 75th percentiles, respectively, whiskers extend to the most extreme non-outlier data points, and outliers are plotted with the red ‘+’ symbol.

In our initial screening to find breath VOCs predictive of COVID infection status, the 108 inputted variables used to develop the PLS-DA models, there were a total of 63 breath VOCs with a variable importance in projection (VIP) score >1, meaning those compounds had an above average influence to discriminate cohorts relative to other compounds. Final PLS-DA models were built using only these 63 compounds. Supplementary Data 2 identifies these compounds and provides mean VIP scores on the final COVID models, the higher the VIP score, the higher the influence of that VOCs in the model classification. Figure 2 and Fig. 3 show the distribution of VOC abundances, normalized to the mean response from non-COVID subjects.

PLS-DA models do not look at individual VOC abundances to discriminate treatment groups, but rather look at a constellation of relative abundances to make the prediction. However, per a Kruskal–Wallis test, twelve compounds had significant differences between at least one pair of treatment groups. Nine compounds (#10, 12, 16, 25, 26, 36, 43, 44, 55) were significant between Delta and Omicron samples. Four compounds (#10, 12, 26, 30) were significant between non-COVID and Delta; and five (#13, 16, 32, 44, 55) between non-COVID and Omicron. See Supplementary Data 2 to map compound numbers to putative identities.

The origin of compounds affected by SARS-CoV-2 (and other respiratory pathogens) in breath is not well understood. What we speculate is that some metabolites can be universal across pathogens or specific to viruses or variants, but we do not know the exact metabolic pathways related to this. They may originate directly from airway cells infected by the virus or may enter the airway through evaporation from blood at the alveolar interface when respiratory gases (oxygen, carbon dioxide) are exchanged with the lungs. Future cell culture studies could reveal the biochemical processes that result in these compounds appearing in exhaled breath.

From our work we make no claims to have developed a breath-based diagnostic test for COVID-19, which would require greater sample numbers, validation, and comparison to other respiratory pathogens (discussed in more detail below). But these findings have important implications towards the development of breath-based viral diagnostics. A pre-print by Sharma et al. of portable device to detect COVID-19 from breath VOCs, accessed in September 2022, concurs with our findings: that models better distinguish COVID from non-COVID breath samples when the Delta and Omicron variants are modeled independently, and that the VOC biomarkers of infection differ between the two variants^21^. The same goes for infections other than SARS-CoV-2^10^. For example, cell culture models of influenza infection showed that viral subtype (H9N2, H6N2, and H1N1) impacted the types and concentrations of emitted volatile compounds^22^. A murine model showed that breath could distinguish the PA01 versus FRD1 strains of *P. aeruginosa*-infected mice^23^.

Other work suggests that some breath biomarkers may not be specific to viral variants. In April 2022, the US FDA approved the InspectIR COVID-19 Breathalyzer under Emergency Use Authorization (EUA) for in vitro diagnostics. In a report made public by the FDA, the sensitivity and specificity are based on samples collected from November 2020 to May 2021 in Colorado, Texas, Louisiana, and Florida^24^. Presumably, these subjects were infected with earlier variants of SARS-CoV-2 as Delta did not surge in the United States until mid-May 2021, according to public data from the US Centers for Disease Control. The InspectIR device was tested against 12 subjects in February 2022 with corresponding sequencing data. Eleven subjects had confirmed Omicron infections, and one did not test positive for SARS-COV-2. Ten were correctly identified as COVID(+) per the breathalyzer, with one false negative. The one negative subject was correctly identified^24^.

A major barrier for breath-based diagnostics has been the inability to validate the selectivity of models against respiratory pathogens other than SARS-CoV-2. In the first 2 years of the pandemic, governments largely adopted masking mandates, social distancing and quarantine/isolation requirements, and stay-at-home orders to prevent transmission of COVID-19. This has caused a major disruption in the global cycle of other respiratory pathogens, and many of them had nearly disappeared until late 2021^25^. Because these breath-based assays measure the host response to infection, it has been challenging, if not impossible, to collect breath samples from individuals infected from other coronaviruses, rhinoviruses, influenzae, etc. Multiplex testing for these organisms is cost prohibitive for certain populations even before the ongoing pandemic as part of patient care^26^. Additionally, COVID-19 related supply chain issues also limited the availability of respiratory pathogen panels in clinical settings.

At the time of writing, however, these viruses have reemerged, providing an opportunity for researchers to validate assays. It is expected that these studies will be published in the upcoming months. While we could not confirm their exact respiratory infection, 90% of non-COVID subjects with respiratory symptoms enrolled into our study were correctly identified as COVID(−) in the Delta+Omicron model and 33% in the Delta-wave model. This true negative proportion increased to 64% in the Omicron wave model.

Still, prior work indicates that breath signatures of infection are specific to pathogens^10^. A cell culture model of primary tracheobronchial epithelial cells showed that models could distinguish volatile emissions of influenza from rhinovirus infections^27^. A study of breath condensate found metabolite differences in breath from those infected with influenza A, human metapneumovirus, and rhinovirus^5^. Breath vapor could distinguish mice infected with one of seven lung pathogens that represent the primary causes of bacterial pneumonia^28^.

## Conclusion

In our work, we reveal that exhaled breath VOCs differ in persons with SARS-CoV-2 depending on the variant of infection. We find that a breath-based diagnostic test may yield higher accuracies if modeling the Delta versus Omicron separately, rather than creating one universal model that does not regard variant. Our findings urge other breath researchers to consider variant as a variable when developing diagnostic tests for SARS-CoV-2 and other respiratory pathogens. More work is needed to compare the selectivity of a potential COVID-19 breath test against other respiratory pathogens, such as influenzae, which had largely disappeared in the first 2 years of the COVID-19 pandemic.

## Supplementary information

Supplementary Data 1

Supplementary Data 2

Supplementary Data 3

Reporting Summary

Description of Additional Supplementary Files

## Data Availability

The datasets generated during and/or analyzed during the current study, including raw mass spectrometry data, are available from the corresponding author on reasonable request. Source data used to plot Figs. 1, 2 and 3 are available in Supplementary Data 3.
