# Supplementary material for: Predominant SARS-CoV-2 variant impacts accuracy when screening for infection using exhaled breath vapor
Source: Commun Med (Lond). 2022 Dec 8;2:158. doi: 10.1038/s43856-022-00221-5 (PMC9731983; doi:10.1038/s43856-022-00221-5)
Supplement: Supplementary file 4 — Reporting Summary [file 43856_2022_221_MOESM4_ESM.pdf]

nature research

Corresponding author(s): Davis, Cristina E

Last updated by author(s): Nov 15, 2022

Reporting Summary

Nature Research wishes to improve the reproducibility of the work that we publish. This form provides structure for consistency and transparency in reporting. For further information on Nature Research policies, see our Editorial Policies and the Editorial Policy Checklist.

Statistics

For all statistical analyses, confirm that the following items are present in the figure legend, table legend, main text, or Methods section.

n/a	Confirmed	
☐	☒	The exact sample size (*n*) for each experimental group/condition, given as a discrete number and unit of measurement
☐	☒	A statement on whether measurements were taken from distinct samples or whether the same sample was measured repeatedly
☐	☒	The statistical test(s) used AND whether they are one- or two-sided<br>*Only common tests should be described solely by name; describe more complex techniques in the Methods section.*
☒	☐	A description of all covariates tested
☒	☐	A description of any assumptions or corrections, such as tests of normality and adjustment for multiple comparisons
☐	☒	A full description of the statistical parameters including central tendency (e.g. means) or other basic estimates (e.g. regression coefficient) AND variation (e.g. standard deviation) or associated estimates of uncertainty (e.g. confidence intervals)
☒	☐	For null hypothesis testing, the test statistic (e.g. *F*, *t*, *r*) with confidence intervals, effect sizes, degrees of freedom and *P* value noted<br>*Give P values as exact values whenever suitable.*
☒	☐	For Bayesian analysis, information on the choice of priors and Markov chain Monte Carlo settings
☒	☐	For hierarchical and complex designs, identification of the appropriate level for tests and full reporting of outcomes
☒	☐	Estimates of effect sizes (e.g. Cohen's *d*, Pearson's *r*), indicating how they were calculated

Our web collection on statistics for biologists contains articles on many of the points above.

Software and code

Policy information about availability of computer code

Data collection	Agilent Chemstation E.02.00.493
Data analysis	Raw mass spectral data was deconvoluted and aligned using Agilent Profinder B.08 and Mass Profiler Professional (MPP, V13.0). Statistical analyses were performed using Mathworks MATLAB R2022a and Eigenvector Research's PLS_Toolbox version 9.0.

For manuscripts utilizing custom algorithms or software that are central to the research but not yet described in published literature, software must be made available to editors and reviewers. We strongly encourage code deposition in a community repository (e.g. GitHub). See the Nature Research guidelines for submitting code & software for further information.

Data

Policy information about availability of data

All manuscripts must include a data availability statement. This statement should provide the following information, where applicable:

- Accession codes, unique identifiers, or web links for publicly available datasets
- A list of figures that have associated raw data
- A description of any restrictions on data availability

The datasets generated during and/or analyzed during the current study are available from the corresponding author on reasonable request.

April 2020

Field-specific reporting

Please select the one below that is the best fit for your research. If you are not sure, read the appropriate sections before making your selection.

☒ Life sciences ☐ Behavioural & social sciences ☐ Ecological, evolutionary & environmental sciences

For a reference copy of the document with all sections, see nature.com/documents/nr-reporting-summary-flat.pdf

Life sciences study design

All studies must disclose on these points even when the disclosure is negative.

Sample size	Sample sizes were contingent on community transmission rates of SARS-CoV-2 at the time of recruitment and collection. As we developed our multivariate analyses in a reiterative way, our sample size of 142 was sufficient, and we report the confidence interval for model accuracies.
Data exclusions	No data were excluded.
Replication	Our COVID models were developed in a reiterative way, with n=50 iterations generated for each model Each iteration contained a calibration and validation set, which samples were randomly assigned to in each iteration.
Randomization	Randomization was not applicable to our study as this was a prospective study.
Blinding	Blinding was not possible for this study as we sought to recruit persons with known SARS-CoV-2 infections.

Reporting for specific materials, systems and methods

We require information from authors about some types of materials, experimental systems and methods used in many studies. Here, indicate whether each material, system or method listed is relevant to your study. If you are not sure if a list item applies to your research, read the appropriate section before selecting a response.

Materials & experimental systems

n/a	Involved in the study	
☒	☐	Antibodies
☒	☐	Eukaryotic cell lines
☒	☐	Palaeontology and archaeology
☒	☐	Animals and other organisms
☐	☒	Human research participants
☒	☐	Clinical data
☒	☐	Dual use research of concern

Methods

n/a	Involved in the study	
☒	☐	ChIP-seq
☒	☐	Flow cytometry
☒	☐	MRI-based neuroimaging

Human research participants

Policy information about studies involving human research participants

Population characteristics	Covariates were not investigated in this study as treatment groups consisted of persons with similar demographic distrutions of age, sex, and race.
Recruitment	We recruited persons aged 18 and older from the UC Davis campus, its medical center and surrounding clinics, and from the greater Sacramento community.
Ethics oversight	Recruitment of volunteers and sample collection was conducted under a previously approved protocol for human subjects research (UC Davis IRB #1636182).

Note that full information on the approval of the study protocol must also be provided in the manuscript.
