# Supplementary material for: Predominant SARS-CoV-2 variant impacts accuracy when screening for infection using exhaled breath vapor
Source: Commun Med (Lond). 2022 Dec 8;2:158. doi: 10.1038/s43856-022-00221-5 (PMC9731983; doi:10.1038/s43856-022-00221-5)
Supplement: Supplementary file 5 — Description of Additional Supplementary Files [file 43856_2022_221_MOESM5_ESM.pdf]

Description of Additional Supplementary Files

File name: Supplementary Data 1

Description: Breath collection dates for all samples analyzed in this work, along with the presumed variant of infection based on our criteria.

File name: Supplementary Data 2

Description: Putative identifications of the 63 breath VOCs used by PLS-DA models (50 iterations each) to discriminate non-COVID from COVID(+) subjects. RT = retention time, MS match factor = reverse match of obtained mass spectra to the NIST 2020 database; KI = Kovats index; VIP = variance in projection score from PLS-DA models (a value indicates the compound had an above average influence to distinguish non-COVID from COVID breath samples in that model).

File name: Supplementary Data 3

Description: Data used to plot Figures 01 and 02.
